# Orange Cookies with Type-4 Resistant Starch: Physical, Nutritional, and Sensorial Characteristics as Evaluated by Patients with Irritable Bowel Syndrome

**DOI:** 10.3390/foods13193144

**Published:** 2024-10-02

**Authors:** Nina G. Heredia-Sandoval, Dulce G. Machado-Duarte, Yolanda M. Preciado-Orozco, Alma R. Islas-Rubio, Ana M. Calderón de la Barca

**Affiliations:** 1 Coordinación de Tecnología de Alimentos de Origen Vegetal, Centro de Investigación en Alimentación y Desarrollo, A.C. Gustavo E. Astiazarán Rosas #46, Col. La Victoria, Hermosillo 83304, Mexico; nina.heredia@ciad.mx (N.G.H.-S.); aislas@ciad.mx (A.R.I.-R.); 2 Coordinación de Nutrición, Centro de Investigación en Alimentación y Desarrollo, A.C. Gustavo E. Astiazarán Rosas #46, Col. La Victoria, Hermosillo 83304, Mexico; dulcegmachado@gmail.com (D.G.M.-D.); yolanda.preciado98@gmail.com (Y.M.P.-O.)

**Keywords:** type 4-resistant starch, cookies, sensorial and nutritional attributes, irritable bowel syndrome

## Abstract

A low-fermentable oligo-, di-, monosaccharide and polyol (low-FODMAP) diet for patients with irritable bowel syndrome (IBS) should include an adequate fiber source. Our aim was to formulate orange cookies using maize flour and type-4 resistant starch (RS4) from maize and to evaluate their properties and sensorial attributes by IBS patients. We prepared two formulations: 37.7% RS4 and 14.7% maize flour and a control with normal maize starch (MS) instead of RS4. We added orange juice and zest instead of water and evaluated their properties. The viscosity, water absorption capacity, and solubility were lower for RS4 than for MS. The width, thickness, L* and a* values of both cookies were comparable (*p* > 0.05), although RS4-C had a decreased b* value and higher hardness (90.6 vs. 80.1 N). The nutrient content was similar between RS4-C and MS-C, but the glycemic index of RS4-C was 63 compared to 95 of MS-C. According to IBS patients, the appearance, taste, hardness, overall quality, and perception of healthiness and nutritional value of both types of cookies were similarly high (*p* > 0.05). Panelists recommend the cookies. Therefore, RS4 cookies could be further investigated for their ability to improve bowel habits and re-equilibrate the microbiota of IBS patients.

## 1. Introduction

Resistant starches (RS) are currently ingredients used by the food industry to improve the technological characteristics of foodstuffs as well as fiber sources. RS are resistant to gastrointestinal digestion and undergo moderate or slow fermentation by microbiota in colon. Two types of commercial RS are type 2 RS (RS2), produced by physical methods, and type 4 RS (RS4), obtained by chemical methods [1], and they both have been studied as important fiber sources for foods of special dietary uses for patients with some gastrointestinal diseases, an emerging area of food technology.

Irritable bowel syndrome (IBS) is a functional gastrointestinal disorder with recurrent abdominal pain and changes in bowel habits, subclassified according to stool types [2]. Symptoms include intolerance to fermentable oligosaccharides, disaccharides, monosaccharides and polyols (FODMAP), principally fructose and fructans (from diverse foods), common in ultra-processed foodstuffs [3]. Therefore, the current treatment for IBS is a low-FODMAP diet, which has shown beneficial effects on symptoms [4]. However, after a few weeks on this treatment, the reduction in dietary fiber from cereals and fruits could lead to increased constipation and a higher dysbiosis index, negatively affecting beneficial microbial species [5].

A low-FODMAP diet including fiber could restore eubiosis in IBS patients. However, non-fermentable fiber is not recommendable for them, while fast fermentable fibers increase the symptoms [3]. Therefore, IBS patients require moderate or slow fermentable fiber without FODMAP, such as resistant starches, to improve intestinal motility and recover eubiosis, with the concomitant production of short-chain fatty acids [6].

In healthy individuals, Deehan et al. [7] and Devarakonda et al. [8] found differential effects of RS4 and RS2, depending on the source, in microbiota and producing butyrate or propionate. In IBS patients in a low-FODMAP diet, RS4 from maize co-administrated with a non-fermentable fiber improved bowel habits [3]. The same dietary treatment increased both the abundance of fermentative bacteria and plasmatic short-chain fatty acids but did not affect those from feces compared to the control [6].

In principle, many gluten-free foods have low-FODMAP content due to the absence of wheat-fructans [9]; however, some contain fruits, vegetables, and legumes with FODMAP [10]. Low-fiber content is common in both gluten-free and low-FODMAP diets, although the latter allows gluten inclusion, facilitating the elaboration of cookies and biscuits with fiber sources for IBS patients. Gluten-free biscuits were prepared using a commercial gluten-free mix plus different levels of RS from sorghum and were sensorially evaluated by healthy individuals [11]. Sahin et al. [9] optimized a model system by adding three different fiber types, primarily RS4 from wheat, into low-FODMAP biscuits made with wheat starch and vital gluten instead of wheat flour; their overall quality was comparable to non-fiber-added low-FODMAP biscuits, according to healthy panelists.

A low-FODMAP diet is difficult to follow, and adherence decreases over long periods [12]. It requires attractive foodstuffs, including bioactive compounds such as dietary fiber, which can go beyond symptoms relief. The preparation of special products for IBS treatment remains a significant challenge, particularly with cereal-based products [13]. Therefore, the aim of this study was to formulate orange cookies using maize flour instead of FODMAP-containing wheat flour with type-4 RS (RS4) from maize and to evaluate their physical, nutritional, and sensorial attributes in patients with IBS.

## 2. Materials and Methods

### 2.1. Materials

Maize resistant starch type 4 (FYBRIN™ RS F100) was supplied by GPC (Grain Processing Corporation, Muscatine, IA, USA). According to the company, it is a cross-linked phosphorylated RS4 resistant starch produced from corn of U.S. origin. Vital gluten was supplied by Mi Granero (ALDAY INGREDIENTES S.A. de C.V., Puebla, Mexico). Normal maize starch (MS) branded as Maizena^®^, nixtamalized maize flour (MASECA^®^), as well as sodium bicarbonate, butter, sugar, eggs, and oranges were purchased from a local market in Hermosillo, Sonora, Mexico. Chemical-grade reagents were obtained from Sigma Aldrich (St. Louis, MO, USA).

### 2.2. Flours and Starches Properties

The swelling power (SP) and solubility (S) of the RS4 and MS were determined according to Bodjrenou et al. [14]. A 0.5 g sample of each starch was mixed with 25 mL of distilled water and heated at 95 °C for 20 min with stirring. The suspension was centrifuged at 1700× *g* for 15 min, and after decantation, an aliquot of the supernatant was evaporated at 105 °C for 2 h. The SP and the S were calculated as follows:

SP (g/g) = Sw/(Ms − S)

where Sw is the sediment weight (g), and Ms is the starch in native mass (g).

S (g/g) = Mds/Ms × 100

where Mds is the dried starch in the supernatant (g), and Ms represents the mass of starch in native form (g).

The water binding capacity (WBC) was evaluated according to the method described by Tang et al. [15]. A 1 g sample was suspended in 5 mL of distilled water and stirred for 30 min at room temperature. The WBC was calculated as follows:

WBC (%) = [(w/w0) − 1] × 100

where w0 (g) is the dry weight, and w(g) is the weight after water absorption.

The viscosity profiles of the flours and starches were carried out using a Rapid Visco Analyzer (RVA-Super4, Newport Scientific, Warriewood, NSW, Australia) according to Method 76-21 [16]. A 3.5 g sample (14% moisture basis) was mixed with 25 g of distilled water. The RVA’s STD1 pasting profile was selected. The test involved heating the sample to 50 °C and holding for 1 min; then, it was brought to 95 °C at the rate of 12 °C/min and was kept at 95 °C for 2.5 min, followed by cooling to 50 °C at the same rate and holding at this temperature for 2 min. Paddle speed was set at 160 rpm. Peak viscosity, trough viscosity, breakdown, setback, and final viscosity parameters were obtained. Analyses were performed in duplicate.

The rapidly digestible (RDS), slowly digestible starch (SDS), total digestible starch (TDS), and resistant starch (RS) contents were evaluated in RS4- and MS starches using the Digestible and Resistant Starch kit (K-DSTRS, Megazyme International, Wicklow, Ireland), according to the manufacturer procedure.

### 2.3. Cookies’ Preparation

Table 1 presents the formulations of each batch for 35 cookies (RS4-C or MS-C). First, fresh orange juice and orange zest were blended and macerated for 30 min at 25 °C before filtering. The blends of starches, maize flour, and gluten were mixed strongly and kept ready. The butter, sodium bicarbonate, and sugar were placed in a kneader (KitchenAid Classic, Benton Harbor, MI, USA), mixed at low velocity for 3 min. Eggs and orange juice were added and mixed for 1 min and then the blend with resistant starch or maize starch, maize flour, and gluten were added and mixed for 3 extra min. Orange juice and zest were used instead of water to achieve the desired dough consistency (43.8 mL for RS4-C and 38.5 mL for MS-C). The doughs were manually molded (approximate thickness = 0.5 cm; dough weigh = 30 g), spread with the mix of juice plus zest and baked in an electric oven (National MFG. Co., Inc., St. Lincoln, NE, USA) at 190 °C for 19 min. Cookies were allowed to cool down for 10 min, and then they were packed in polyethylene bags and kept at room temperature.

### 2.4. Cookies’ Physicochemical Properties and Nutritional Composition

The proximal analysis of the cookies was carried out according to AACC [14] Methods 44.15.01, 46-13.01, 30.20.01, and 08.01.01 for moisture, protein (N × 5.6), fat, and ash, respectively. The fructose content of both RS4-cookies and MS-cookies was quantified by high performance liquid chromatography (Thermo Scientific, Dionex UltiMate 3000, Waltham, MA, USA) with a refractive index detector (Refracto Max 520, ERC Inc. Kawaguchi, Saitama, Japan) using an amino column for separation [17].

Cookie width and thickness were determined following the AACC Method 10-50D [14]. Cookie hardness was measured with a texturometer (Model TA-XT2, Stable Micro System, Godalming, Surrey, UK) using a knife blade (thickness = 0.3 cm). Cookies placed in an adjustable three-point bend assembly were traversed using a test speed of 1 mm/s and a distance of 7 mm. The results reported are the mean of the maximum force for each cookie formulation. Cookie crust color, values L*, a*, and b* were measured using a Minolta colorimeter ((Model Cr 400, Minolta Co., LTD., Osaka, Japan) with a white tile as a reference. Cookie hardness, color, and moisture were recorded at days 0, 1, and 5 after baking and storage at room temperature.

### 2.5. Starch Fractions and Glycemic Index

The digestible, resistant and total starch contents were evaluated in RS4- and MS-containing cookies using the Resistant Starch Assay kit (K-RSTAR, Megazyme International, Wicklow, Ireland), according to Method 32-40.01 [16]. The glycemic index (GI) in vitro was determined [18]. The hydrolysis index (HI) was the ratio between the areas under the curve of the sample and the reference food (white bread). The GI was estimated as [19] using the equation GI = 39.71 + 0.549 (HI).

### 2.6. Sensory Evaluation

The panelists were 20 patients (22 to 42 years old) diagnosed with IBS by gastroenterologists at least 2 years prior. Among them, 9 were classified as constipation-predominant, 3 as diarrhea-predominant, 5 as mixed, and 3 as unknown according to the Rome IV criteria [2]. The patients’ intolerances included fructans (13 of them), lactose and sorbitol (12 of them), and fructose (8 of them). Hedonic tests were performed for each cookie formulation using a 1–10 scale, where 1 represented the least accepted and 10 the most accepted value. After consuming the cookies daily for 4 weeks, panelists evaluated the appearance, texture, flavor, and overall acceptability, and questions about their perception of the cookies’ quality were related to health and nutrition. Additionally, they answered questions about any complications or discomfort experienced after consumption, and whether they would recommend the cookies (scale −2 to 2). Before the assays, participants signed the informed consent as part of the registered protocol by our institutional Ethics Committee of Research with the code: CEI/023-1/2022. 

### 2.7. Statistical Analysis

Differences were determined by a one-way analysis of variance (ANOVA) for the cookies’ physicochemical and nutritional properties. For texture, moisture, and color at different storage times, a 3 × 2 factorial experiment arranged in a completely randomized design was used, considering storage time (0, 1, and 5 days) as factor 1 and the two treatments (RS4-C and MS-C cookies) as factor 2. Multiple comparisons of the means were performed using the Tukey–Kramer test when interactions were not significant. For sensory analysis, the values were expressed as median and interquartile range, and the U-test for non-parametric data was used. Values with a *p* < 0.05 were considered statistically significant. Statistical analysis was conducted using NCSS software (version 2007).

## 3. Results and Discussions

### 3.1. Starch and Flour Properties

Digestibility fractions, swelling power, solubility, and water absorption of the two used starches RS4 and MS are presented in Table 2. As expected, more than 97% of the RS4 was resistant starch, in contrast to a similar percentage of MS as digestible starch (96.8%). Therefore, the swelling properties and solubility of RS4, with very low values, were lower than those of MS (*p* < 0.05); thus, the water binding capacity of RS4 was also significatively lower. After the modification by crosslinking, the starches keep the granule integrity and their breakdown is slow; in addition, the induced intramolecular and intermolecular bonds between linear amylose and branched amylopectin chains limit swelling [14,20]. According to Korkut and Kahraman [21], the modification by crosslinking with phosphates, as in the RS4 we included, stabilizes the starch granules against shear force, decreases swelling power and affects their pasting behavior. Additionally, we used in our formulations another starch source, the nixtamalized maize flour, which contains 6.26% dietary fiber and has a total digestible starch content of less than 60% [22].

The viscosity profile (Table 3 and Figure S1 in Supplementary Materials) shows that the incorporation of RS4 resulted in pasting curves at 14% solids that did not rise above the baseline when heated from 50 to 95 °C. In contrast, native MS and their blends with maize flour and gluten exhibited regular pasting curves. Consequently, MS presented a higher peak viscosity and final viscosity values, attributable to a proportion of free starch granules and an increase in peak viscosity with decreasing particle size [23]. Additionally, the particle size in the blend of MS with maize flour and gluten was apparently coarser. The peak, final viscosity, and the setback between the MS and MS-Blend showed a significant reduction (~70%) in these parameters. The peak viscosity indicates the point of maximum swelling of starch granules, and the presence of protein in the MS-Blend may affect the water uptake by starch granules, resulting in a lower peak viscosity. Also, proteins could affect amylose leaching and limit the reorganization process, modifying the final viscosity and setback [24]. Table 3 also shows no significant increase in viscosity for RS4-Blend compared to RS4 alone (*p* > 0.05); only the final viscosity was different (*p* < 0.05). The viscosity measurement is reliable for inferring the type and degree of crosslinking of the starch; those with higher crosslinking exhibit a significant reduction in peak viscosity, as it was shown in the flat curve of the RS4. According to the swelling power and the RVA profile, it is possible to infer a high degree of crosslinking of the RS4 used in our study. Such crosslinking inside of starch granules hardened the structure and induced a diffusional resistance to water percolation, which reduced granule swelling and drastically modified the pasting curves and fluidity [21,25].

### 3.2. Cookies’ Description

The RS4-containing cookies (RS4-C) and the MS-containing cookies (MS-C) were practically indistinguishable (*p* > 0.05) from each other in dimensions. These width (55.5 ± 0.4 vs. 56.0 ± 0.6) and thickness (20.6 ± 0.5 vs. 21.0 ± 0.1) for RS4-C and MS-C, respectively, were comparable. These dimensions are different from those expected to obtain with the formulation of the method 10-50D [16]. Cookie components may behave differently after mixing together. When the various ingredients in the dough are added, the processing parameter must also be adjusted, as it was performed with the SR4-Blend and the MS-Blend. The addition of RS4 to the cookie formulation did not substantially affect their physical characteristics, which is consistent with previous studies [11]. However, the addition of RS4 to a biscuit preparation caused shrinkage during baking [9]. In the last study, the biscuits were prepared with wheat starch and RS4 from wheat plus other fiber sources, which differs from our formulation.

**Figure 1:**
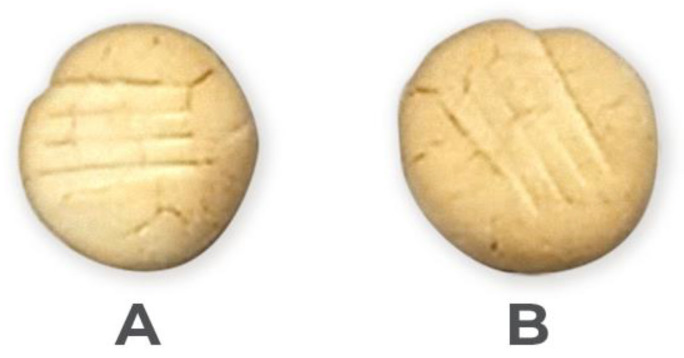
Digital image of RS4-containing (**A**) and MS-containing (**B**) orange cookies.

The appearance of the RS4-containing and MS-containing orange cookies is shown in Figure 1. Both types of cookies were visually similar, with some cracks on the surface, although the RS4-cookies exhibited more homogeneity. Generally, a cracking in the cookies is due to rapid moisture loss from the surface, which is replaced by water diffusing from the center of the cookies.

### 3.3. Physicochemical Properties of the Cookies

The hardness and color characteristics of the fresh RS4-C and MS-C are presented in Table 4. The RS4-C had a significantly higher (*p* < 0.05) hardness than the MS-C cookies. RS4 starches are known to recrystallize faster than digestible starch due to differences in morphology [9]. Thus, the faster crystallization rate between granules of RS and digestible starch could increase the hardness of the RS4-containing cookies. The color of the cookies mainly depends on the type of flour used and is a crucial parameter for consumer acceptability. The MS-containing cookies were slightly darker than the RS4-containing cookies, as indicated by lower L* values. This difference can be attributed to the higher protein and reducing sugar contents in the MS-C, which induces a browning reaction in the cookies. The difference between yellowness (b*) values was statistically significant (*p* < 0.05) due to the extra nixtamalized maize flour in the formulation.

The physicochemical characteristics of both types of cookies, such as moisture, color, and hardness, were measured after 1 and 5 days of storage at room temperature. The effects of the sample, storage time, and interactions were calculated. Regarding cookie moisture at different storage times, no significant interaction of the main effects was found. The moisture content was lower in RS4-C than in MS-C, which can be attributed to the low water-holding capacity of RS, contributing to the extended shelf life of foods [6]. The hardness of the RS4-C remained consistent (90.63 ± 5.57 N and 90.30 ± 10.66 N) from 0 to 5 days, while the hardness of the MS-C increased from 80.12 ± 2.95 N to 103.41 ± 2.17 N, with significant differences (*p* < 0.05) due to the type of sample. Respect to color, the L* value increased over the storage days, while the a* and b* values decreased. Despite the significance due to interactions between the type of sample and storage time, the differences were small over the 5 days.

### 3.4. Nutritional Properties of the Cookies

Regarding the nutritional quality of the RS4-containing cookies, Table 5 shows the nutrient content, hydrolysis index, and glycemic index in comparison to the MS-cookies. The moisture and protein contents were lower (*p* < 0.05) in the RS4-cookies than in the MS-C. The moisture results align with the water binding capacity, where the native MS shows a better capacity to bind water than the RS4. The protein content was higher in the MS-C due to a higher content of maize flour in its formulation. The inclusion of RS4 in the formulation caused an increase (*p* < 0.05) in the ash content of cookies, likely due to the crosslinking of the starch with a phosphorous-containing agent, which increased the mineral content. The lipid content was similar in the RS4- and MS-containing orange cookies.

We found that each cookie with 10 g of RS4 contained 30.5 mg of fructose and the cutoff value to consider low FODMAP is 150 mg per serving. Orange is one of the fruits recommended for low-FODMAP diets due to its low content of fructose [26]. We used orange juice because the low-FODMAP diet adherence decreases over long periods and patients require attractive foodstuffs, and the orange taste and aroma are highly acceptable as it was evaluated (results in Table 6).

As expected, the RS4-containing cookies presented significantly (*p* < 0.05) lower values of hydrolysis (100.51 vs. 42.57) and a glycemic index than the MS-containing cookies (94.89 vs. 63.08). This result is related to the less digestible and higher RS content in the RS4-C than in the MS-C. The RS4-cookies had significantly less (*p* < 0.05) digestible starch (40.7 ± 0.9 vs. 56.5 8 ± 2.5) and more RS (12.6 ± 0.1 vs. 0.81 ± 0.0) than the MS-cookies. The degree of crosslinking of RS4 influences the enzymatic breakdown of starch due to the inaccessibility of the enzyme because of steric hindrance, resulting in lower glucose release and a lower glycemic index [14]. Although our primary interest in including RS4 in the cookies for patients with IBS was to provide a fiber source, it is important to have a product with a medium–low glycemic index. This necessity is because the doses of the prepared cookies could be up to three or four pieces per day, over 2 or 3 months, depending on the fiber content of the patient’s diet. In addition to its effects as a dietary fiber not digested in the small intestine but fermented in the large intestine, RS4 plays a significant role in glucose homeostasis. Therefore, RS4 could be indicated for patients with type 2 diabetes [1].

Finally, the dietary treatment for IBS patients is a low-FODMAP diet that contains the required nutrients if well formulated [4], except by its low fiber content. This is because the low-FODMAP excludes several fruits and vegetables as well as wheat and related cereals. Therefore, patients need an extra fiber source; it should be low-FODMAP itself and have slow fermentation properties to avoid triggering symptoms. Therefore, the fiber source included in cookies do not need to have high nutritional quality, but they do need at least a medium glycemic index so that its daily intake can preserve glucose homeostasis.

### 3.5. Sensorial Characteristics of the Cookies

The results for the sensorial analyses of the cookies are shown in Table 6. There were no significant differences between the RS4-C and MS-C for any of the attributes evaluated, including appearance, texture, flavor, and overall acceptance, with interquartile range (IQR) values from 8 to 10 for both types of cookies (*p* > 0.05). The median values were high (9.5–10) except for texture (8.5 vs. 9) for the RS4-C and MS-C, respectively, but with no significant differences (*p* > 0.05); the texture could be attributable to the cookies’ hardness. High palatability values could be related to the content of butter, sugar, and orange juice with zest, which enhance the flavor and aroma. It is also known that shortening and sugars greatly affect flavor, sweetness, mouthfeel, and texture in cookies [27]. Similarly, no significant differences (*p* > 0.05) were observed in the perception of healthiness or nutritiousness between the RS4-cookies and MS-cookies by the patient panelists. Opinions of IBS patients indicated their interest for recommending the cookies; they were satisfied with the consumption, which required more liquid intake as the cookies were consumed with water, coffee, or other beverages.

## 4. Conclusions

Our general objective was to formulate orange cookies with a low-FODMAP content based on maize flour, vital gluten, and RS4 as a fiber source, which were chosen as the best ingredients and to evaluate their physical, nutritional, and sensorial properties.

More than 97% of the RS4 was resistant starch in contrast to a similar percentage of MS as digestible starch. With respect to the flour blends including RS4 or MS, the experiment resulted in a viscosity measurement reflecting the high degree of crosslinking of the RS4 granules, which exhibited a significant reduction in peak viscosity and granule swelling, modifying the pasting curves and fluidity.

The addition of RS4 to the cookie formulation did not affect their physical characteristics; both RS4-cookies and the MS-cookies were visually similar. The hardness was higher for the RS4-cookies than for the MS-cookies; however, the low water binding capacity of the RS4-cookies contributed to extending their shelf life, maintaining the same hardness after 5 days in storage, while that of the MS-cookies increased.

With respect to the nutritional quality, it is important to consider that the RS4-containing cookies were designed for a special dietary treatment. Our primary objective in including RS4 in the cookies for patients with IBS was to provide a high fiber content with slow fermentation. RS4’s resistance to intestinal hydrolysis induced an important effect for reduction in the glycemic index, which is important for a product intended for daily consumption to maintain glucose homeostasis. The content of lipids, protein, and sugar of the RS4-cookies were similar or better than the content of commercial similar cookies but without any additives for preservation.

The most interesting properties of the produced RS4-containing orange cookies were their sensorial characteristics. They had a high acceptability and a high perception of healthiness and nutritional value by the patients with IBS, even after one month of daily consumption. Their recommendation of the orange RS4-cookies was high, and their tolerance was acceptable. An adherence to the dietary treatment of IBS is difficult; thus, if the patients liked the RS4-containing orange cookies for one month, the possibility to include it in a low-FODMAP diet could be high.

Finally, it is still necessary to test if the addition of RS4 to the orange cookies might alleviate constipation and positively modify the microbiota of IBS patients.

## Figures and Tables

**Table 1:** Formulation of the orange cookies with maize flour plus type-4 resistant starch (RS4-C) or maize starch (MS-C).

	Weight (g)	
Ingredients	RS4-C	MS-C
Resistant maize starch	393.8	-
Maize starch	-	349.9
Maize flour	152.1	195.9
Sugar	196.9	196.9
Dry gluten	32.8	32.8
Sodium bicarbonate	6.1	6.1
Butter	131.3	131.3
Egg	131.3	131.3

**Table 2:** Digestible fractions and physical properties of starches RS4 and MS *.

Starch	Rapid Digested	Slow Digested	Resistant	Swelling Power	Solubility	Water Binding Capacity
	(%)					
RS4	0.28 ± 0.11 ^a^	1.63 ± 0.54 ^a^	97.46 ± 0.11 ^b^	3.40 ± 0.13 ^a^	1.16 ± 0.13 ^a^	77.40 ± 6.93 ^a^
MS	24.49 ± 0.45 ^b^	72.28 ± 0.3 ^b^	3.22 ± 0.15 ^a^	15.03 ± 0.07 ^b^	13.64 ± 0.17 ^b^	87.37 ± 5.25 ^b^

* Values with different letters within the same column are significantly different (*p* < 0.05).

**Table 3:** Viscosity profile parameters of starches (RS4 and MS) and the blends of starches with maize flour and gluten (RS4-Blend and MS-Blend) *.

Sample	Peak Viscosity	Trough Viscosity	Breakdown	Setback	Final Viscosity
			(cP)		
RS4	13.0 ± 0.0 ^a^	10.5 ± 0.7 ^a^	2.50 ± 0.7 ^a^	1.5 ± 0.7 ^a^	12.0 ± 1.4 ^a^
MS	3180 ± 155.6 ^c^	2293.5 ± 0.0 ^c^	886.5 ± 68.6 ^c^	1435 ± 50.9 ^c^	3728.5 ± 137.9 ^d^
RS4-Blend	41.5 ± 0.7 ^a^	41.5 ± 0.7 ^a^	1.0 ± 0.0 ^a^	10 ± 0.0 ^a^	51.5 ± 0.7 ^b^
MS-Blend	833.5 ± 9.2 ^b^	726.0 ± 8.5 ^b^	107.5 ± 0.7 ^b^	393 ± 0.0 ^b^	1119 ± 8.5 ^c^

* Values with different letters in the same column are significantly different (*p* < 0.05).

**Table 4:** Hardness and color characteristics of RS4-containing (RS4-C) or maize starch-containing (MS-C) orange cookies.

Sample	Hardness (N)	Color		
		L*	a*	b*
RS4-C	90.63 ± 5.57 ^b^	73.36 ± 1.60 ^a^	5.19 ± 0.91 ^a^	34.43 ± 1.44 ^a^
MS-C	80.12 ± 2.95 ^a^	71.45 ± 1.43 ^a^	6.57 ± 0.78 ^a^	38.07 ± 0.55 ^b^

Values are means of quadruplicate ± standard deviation. Values with different letters in the same column are significant different (*p* < 0.05).

**Table 5:** Nutrients of RS4-containing (RS4-C) or maize starch-containing (MS-C) orange cookies.

Sample	Moisture	Protein	Lipids	Ash	Carbohydrates *	HI	GI
	(%)						
RS4-C	8.25 ± 0.25 ^a^	6.28 ± 0.29 ^a^	12.17 ± 0.22 ^a^	1.23 ± 0.04 ^b^	72.07	42.57 ^a^	63.08 ^a^
MS-C	8.65 ± 0.03 ^b^	6.93 ± 0.04 ^b^	12.36 ± 0.25 ^a^	0.89 ± 0.00 ^a^	71.17	100.51 ^b^	94.89 ^b^

* Calculated by difference. Mean value of triplicates ± SD. Different letters in the same column indicate statistical difference (*p* < 0.05); HI, hydrolysis index; GI, glycemic index.

**Table 6:** Sensory evaluation of orange RS4 or MS-cookies made with maize flour and maize starches RS4 or MS, (*n* = 20) ^4^.

Attribute	RS4-Cookies	MS-Cookies	*p*-Value
Appearance ^1^	10 (9–10)	10 (8–10)	0.565
Texture ^1^	8.5 (6–9)	9 (8–9)	0.263
Flavor ^1^	9.5 (8–10)	10 (9–10)	0.161
Overall acceptance ^1^	9.5 (8–10)	9.5 (9–10)	0.804
Healthy ^2^	9 (8–10)	9 (8–10)	0.922
Nutritious ^2^	9 (8–10)	9 (8–10)	0.768
Recommendation ^3^	2 (1–2)	2 (1–2)	0.551
Tolerance ^3^	0.5 (−1 to 1)	1 (0 to 2)	0.461

^1^ Scoring system: 1 (dislike extremely) to 10 (like extremely) on the appearance, texture, flavor, and overall acceptance of the cookie’s; ^2^ Scoring system: 1 (not at all healthy or nutritious) to 10 (extremely healthy or nutritious); ^3^ “I tolerated the cookie well, with no complaints” by marking rating of −2 (Strongly disagree), −1 (disagree), 0 (no opinion), 1 (agree) and 2 (strongly agree). Values were expressed as median and interquartile range (IQR); ^4^ The U-test for non-parametric data was used.

## Data Availability

The original contributions presented in the study are included in the article and Supplementary Materials, further inquiries can be directed to the corresponding author.

## Supplementary Materials

The following supporting information can be downloaded at: https://www.mdpi.com/article/10.3390/foods13193144/s1, Figure S1: Viscosity profile of type-4 maize starch (RS4) and maize starch (MS), and their respective blends with maize flour and gluten (RS4-B and MS-B).
