# Supplementary material for: Orange Cookies with Type-4 Resistant Starch: Physical, Nutritional, and Sensorial Characteristics as Evaluated by Patients with Irritable Bowel Syndrome
Source: Foods. 2024 Oct 2;13(19):3144. doi: 10.3390/foods13193144 (PMC11476083; doi:10.3390/foods13193144)
Supplement: Supplementary file 1 [file foods-13-03144-s001.zip › foods-3175164-supplementary.pdf]

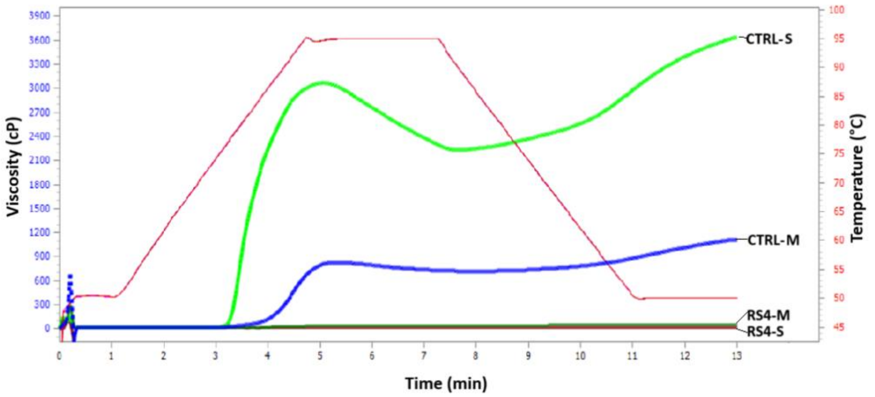

Figure S1. Viscosity profile of type-4 maize starch (RS4-S) and commercial maize starch (control) (CTR-S), and their respective mixes with maize flour and gluten (RS4-M and CTR-M).
